# Validation of a semiautomated system for surveillance of surgical site infection after cesarean section

**DOI:** 10.1017/ice.2021.264

**Published:** 2022-10

**Authors:** Pnina Shitrit, Ravid Mudrik, Bat-Sheva Gottesman, Michal Y. Chowers

**Affiliations:** 1 Infection Control Unit, Meir Medical Center, Kfar Saba, Israel; 2 Sackler Faculty of Medicine, Tel-Aviv University, Tel-Aviv, Israel; 3 Infectious Diseases Unit, Meir Medical Center, Kfar Saba, Israel

## Abstract

Surveillance of surgical site infection after cesarean section is challenging due to the high volume of these surgeries. A manual chart review of women undergoing cesarean section between January and June 2017 (675 charts, 40 infections) was compared to charts identified via an algorithm (141 charts, 39 infections). The algorithm achieved 97.5% sensitivity and 83.9% specificity and reduced the workload of infection control personnel.

Surgical site infection (SSI) surveillance is an essential tool for infection control. SSI rate is an important indicator of quality of care. Comparing institutional rates to a benchmark and providing feedback to surgeons have been shown to reduce infection rates.^1^

Complete chart review is a commonly used surveillance method; it can be done retrospectively, and it is effective.^2^ Chart review is less desirable for high-volume surgeries, such as cesarean section, because it is very time-consuming for infection control personnel (ICP). Thus, more efficient methods are needed, such as electronic surveillance systems,^3^ which rely on data collected automatically from hospital databases. In a semiautomated surveillance system, an algorithm identifies cases with high probability of infection, and subsequently, ICP verify or rule out infection based on manual chart review of identified cases. To reduce workload, an ideal semiautomated system would have high sensitivity with high negative predictive value (NPV) (ie, not missing infected individuals) and high specificity. Several electronic algorithms have been validated for various surgeries,^4,5^ but the patient population undergoing cesarean section is unique and thus it merits a specific algorithm. Our objective was to validate a semiautomated system for cesarean section SSI surveillance.

## Methods

We conducted a validation study on an historical cohort. The setting was a secondary-care, 740-bed, university-affiliated hospital in central Israel, with ∼6,000 births and ∼1,300 cesarean sections per year. In this study, we reviewed the electronic medical records of all women who underwent cesarean section between January 1 and July 30, 2017.

All electronic medical records of study participants were reviewed retrospectively by one of the authors (R.M.) to assess the presence of SSI based on US Center for Disease Control and Prevention (CDC) definitions.^6^ Variables collected included demographics, gestational age at delivery, body mass index (BMI), comorbidities, previous cesarean section, antibiotic prophylaxis, cesarean section duration, American Society of Anesthesiologists (ASA) score, rupture of membranes, imaging, postoperative antibiotics, bacteriology results, wound class, diagnosis at discharge, length of hospital stay (LOS), readmissions or visits to the emergency department (ED) up to 30 days after cesarean section, ambulatory visits to family physician, and antibiotic prescription in the community within 30 days after cesarean section.

In parallel, an algorithm was built to seek suspected cases for further manual review of the medical records by hospital ICP. The algorithm included at least 1 of the following criteria: (1) LOS >4 days, (2) fever during labor, (3) rehospitalization or return to any ED within 30 days of cesarean section, (4) blood or wound cultures performed during hospitalization, and (5) any antibiotic given during hospitalization, excluding prophylaxis. In all suspected cases, SSI was diagnosed independently by ICP according to CDC definitions.^6^

Because this was a quality intervention, it was not subject to approval by the institutional review board nor did it require written informed consent.

### Statistical analysis

Categorical variables were described as percentages and numerical variables as mean or median with standard deviation (SD) or range, as appropriate. For the validation phase, the complete chart review was used as the gold standard and the semiautomated surveillance system was compared to it. Sensitivity, specificity, positive predictive value (PPV), and NPV were calculated. These parameters were calculated for the entire tool and for each separate component.

## Results

During the study period, 675 patients underwent cesarean section. The median age was 33 years (range, 18–53). The median BMI was 27.2 (range, 16.9–57.2), and the mean gestational age at delivery was 37.8 ± 2.8 weeks. The most common comorbidity was diabetes (9%), and 7% of the cohort smoked tobacco. The median duration of surgery was 39 minutes (interquartile range, 32–50); 61% were emergency operations; and most (88%) were clean/contaminated operations. The most common indication for surgery was “nonreassuring fetal heart rate.”

Among the 675 surgeries, the complete chart review identified 40 SSIs (5.9%). Among them, 29 (4.3%) were superficial incisional SSIs, 1 (0.1%) was a deep incisional SSI, and 10 (1.5%) were organ-space SSIs.

The automated surveillance method identified 141 suspected cases, 21% of the cohort. Among these, chart review identified 39 infections: 27% of the suspected cases and 5.6% of the entire cohort. The sensitivity of the semiautomated surveillance system was 97.5% (95% confidence interval [CI], 87.1–99.5); specificity was 83.9% (95% CI, 80.9–86.7); PPV was 27.6 (95% CI, 20.9–35.5); and NPV was 99.8% (95% CI, 98.9–99.9) (Table 1). One case of superficial infection that was identified and treated in the community was missed.

**Table 1.** Results of the Electronic Surveillance Method

Variable	Suspected	True Positive	Sensitivity	Specificity	PPV	NPV
Model with ≥1 of the following variables	141	39	97.5	83.9	27.6	99.8
ED within 30 d	83	34	85.0	92.3		
LOS >4 d	25	9	22.5	97.4		
Fever at birth	22	2	5.0	96.8		
Antibiotics	56	37	92.5	97.0		
Blood culture	31	21	52.0	98.4		
Wound culture	28	26	65.0	99.6		
Model with 1 of 2 variables (LOS>4 d, return to ED)	107	39	97.5	89.3	36.4	99.8

Note. PPV, positive predictive value; NPV, negative predictive value; ED, emergency department; LOS, length of hospital stay.

Based on the electronic surveillance, all of the identified cases either had an LOS >4 days or the patient returned to the OBGYN ED within 30 days of the cesarean section. Limiting the electronic tool to these 2 identifiers would have reduced the number of suspected cases to 107 (16%) without missing any, thus increasing specificity to 89.3% and reducing ICP workload.

## Discussion

Cesarean delivery is a common procedure, accounting for >30% of births in the United States^7^ and >20% in our medical center in Israel. These numbers make manual SSI surveillance with chart review incredibly challenging. In this study, we validated a semiautomatic tool that reduced ICP workload by 79% and had 97.5% sensitivity, missing only 1 patient with a superficial wound who did not return to the hospital.

Patients undergoing cesarean section are typically healthy young women without comorbidities. Moreover, hospitalization after surgery is short and standardized. When a young woman returns to the ED after delivery, the visit is usually related to the delivery itself. As in other studies, we collected data on comorbidities, smoking status, duration of surgery, physician diagnosis, antibiotic use and more, but none of these data added sensitivity to our tool. In most cases, either the infection was evident early, causing prolonged hospitalization, or was evident late, resulting in return to the ED. The only case missed was a mild, superficial infection treated in the community, which would have been missed by any tool built on hospital-based data. Notably, the tool we created to identify cesarean section SSIs is specific to this patient population and would not be appropriate for an elderly, sick population undergoing orthopedic or colon surgeries.

Few studies have specifically investigated an electronic tool for SSIs after cesarean section. Studies by Miner et al^8^ and Yokoe et al^9^ screened automated, ambulatory administrative claim data that were used to identify cesarean-section SSIs. The indicators they identified were diagnosis of infection, blood and wound culture, and antibiotics.^8,9^ The specific electronic database used should be tailored to each health system. Considering the high volume of cesarean section surgeries and the constraints in ICP personnel, an automated tool is essential for timely and efficient surveillance of cesarean-section SSIs.

This study has several limitations. It was a single-center study; the semiautomated system would miss patients who present for postoperative care at another hospital. Furthermore, in other countries, the LOS after cesarean section or the barriers to return to the ED might be different. However, compared to patients undergoing other surgeries, postdelivery women are a homogeneous population that require a focused approach. Our gold standard for SSI diagnosis was the complete chart review method and not prospective follow-up of patients, which is nearly impossible with such a high-volume surgery.

In conclusion, this study validates a semiautomated tool with high sensitivity and high specificity that identifies SSIs after cesarean section. It can help provide valuable information and decrease the workload for ICP.
